# lncRNA MIR4435‐2HG promoted clear cell renal cell carcinoma malignant progression via miR‐513a‐5p/KLF6 axis

**DOI:** 10.1111/jcmm.15609

**Published:** 2020-07-30

**Authors:** Kai Zhu, Chenkui Miao, Ye Tian, Zhiqiang Qin, Jianxin Xue, Jiadong Xia, Shenhao Zhu, Aiming Xu, Jie Yang, Zengjun Wang

**Affiliations:** ^1^ Department of Urology The First Affiliated Hospital of Nanjing Medical University Nanjing China; ^2^ Department of Urology The Second Hospital of Nanjing Nanjing University of Chinese Medicine Nanjing China; ^3^ Department of Urology Nanjing First Hospital Nanjing Medical University Nanjing China

**Keywords:** ccRCC, invasion, KLF6, long non‐coding RNA, MIR4435‐2HG, proliferation

## Abstract

Long non‐coding RNAs (lncRNAs) take various biological effects in clear cell renal cell carcinoma (ccRCC) mostly through sponging with microRNAs (miRNAs). lncRNA MIR4435‐2HG is found to promote tumour progression in gastric cancer, glioblastoma and hepatocellular carcinoma. However, the role of lncRNA MIR4435‐2HG in ccRCC progression remains unknown. The purpose of this research was to investigate the potential molecular mechanism of lncRNA MIR4435‐2HG regarding the regulation of ccRCC initiation and progression. In this study, we found the up‐regulation of MIR4435‐2HG in ccRCC tissues and cell lines. Functionally, overexpression of MIR4435‐2HG promoted the proliferation as well as the metastasis in ccRCC cell lines, whereas knockdown of MIR4435‐2HG inhibited the above changes. Then, bioinformatic analysis and luciferase reporter assays confirmed the negative regulation effect of MIR4435‐2HG on miR‐513a‐5p. And further investigations showed that KLF6, which collected from the intersection of databases, was the potential conjugated mRNAs of miR‐513a‐5p. Finally, the rescue experiments revealed the relation among MIR4435‐2HG and KLF6, which showed that KLF6 could reverse the promoting effect of MIR4435‐2HG on ccRCC in vitro and in vivo. Therefore, our findings provided insight into the mechanisms of MIR4435‐2HG in ccRCC and revealed an alternative target for the clinical diagnosis and treatment of ccRCC.

## 1 INTRODUCTION

Globally, renal cell carcinoma (RCC) is one of the most common malignancies in urinary system and accounts for approximately 4% of adult malignancies.^1^, ^2^ Clear cell RCC (ccRCC), which derives from the epithelial cells of the proximal renal tubule, accounts for 75%‐80% of RCC.^3^ Currently, the treatment of ccRCC patients remains far from satisfactory, because of radiotherapy and chemotherapy resistance. Thus, tumour resection is the best choice of treatment for ccRCC patients, which is considered the only potential treatment that results in a complete cure.^4^ Normally, most ccRCC patients are found during the advanced stage, because of occult onset and fast progression.^5^ The multifactorial and complexity nature of the proliferation mechanism of ccRCC is an intricate network involving multiple carcinogens and varying genetic backgrounds, which lead to the alterations of tumour suppressors or oncogenes.^6^ Therefore, it is necessary to identify the molecular mechanisms of ccRCC progression, which is useful in diagnosing and treating.

Recent findings have uncovered the transcription of long non‐coding RNAs (lncRNAs) from human genome with the development of high‐throughput sequencing.^7^, ^8^, ^9^ lncRNAs, which are more than 200 nucleotides long, have no or limited protein‐coding capacity.^10^ And lncRNAs can be classified into five types including sense, antisense, intergenic, bidirectional and intronic according to its location.^11^ Emerging evidence has documented that lncRNAs were identified to play diverse roles as a molecular modulator in regulating various physiological and pathological processes, such as organ development, immunity, tumorigenesis and tumour progression.^12^, ^13^, ^14^ Previous studies revealed that lncRNAs exerted their roles in all aspects of gene regulation and protein function, such as tethers, decoys, guides and scaffolds.^15^, ^16^, ^17^, ^18^ Nowadays, increasing studies have reported that a number of lncRNAs might play multiple roles in the initiation and progression of ccRCC.^19^, ^20^ MIR4435‐2HG, which reported with aberrant expressions in some malignant tumours, could regulate tumorigenesis and progression.^21^, ^22^, ^23^ Meanwhile, we found the abnormal expression of MIR4435‐2HG in ccRCC by TCGA data analysis. Nevertheless, the mechanisms and functions of MIR4435‐2HG in ccRCC were poorly known. Therefore, we aimed to investigate the potential molecular mechanism of lncRNA MIR4435‐2HG in cell proliferation and invasion of ccRCC.

## 2 MATERIALS AND METHODS

### 2.1 Clinical specimens

A total of 40 matched samples of ccRCC specimens and adjacent non‐cancerous tissues were obtained for this investigation. None of the patients had received chemotherapy or radiotherapy before surgery. All the samples were frozen immediately in liquid nitrogen after surgical resection and stored at −80°C until further analysis (Table 1). The clinical and pathological characteristics were obtained from our hospital. The Institutional Review Board approved the use of the tumour samples and animals in this study.

### 2.2 Cell culture

The human ccRCC cell lines (786‐O, 769‐P, Caki‐1, Caki‐2, ACHN and A498), human renal tubular epithelial cell line (HK‐2) and human embryonic kidney (HEK) 293FT cell line were maintained by our laboratory. All cells were cultured according to protocols recommended by the American Type Culture Collection (ATCC, Manassas, VA, USA). In addition, all cell lines were cultured in a standard humidified atmosphere with 5% CO_2_ at 37°C.

### 2.3 In situ hybridization

Paraffin‐embedded sections of ccRCC tissues were deparaffinized with xylene and rehydrated with 100, 90, 70 and 50% ethanol (5 minutes each) at room temperature. The samples were digested with proteinase K and fixed in 4% paraformaldehyde for 10 minutes at room temperature, followed by hybridization with the MIR4435‐2HG probe (Servicebio, Wuhan, China) at 55°C overnight and subsequent incubation with HRP‐conjugated secondary antibody for 30 minutes at 4°C. Diaminobenzidine was used to develop the stain with a colorimetric reaction for 30 minutes at room temperature, and then, the sections were observed under light microscope.

**Table 1:** Relationship of MIR4435‐2HG expression and clinicopathological characteristics of the 40 ccRCC patients

Variables	Patient number (N)	MIR4435‐2HG expression		*P*‐value	Chi‐square
		Low (n = 19)	High (n = 21)		
Age (years)				0.516	0.4224
≥60	19	8	11		
<60	21	11	10		
Gender				0.554	0.3510
Female	17	9	8		
Male	23	10	13		
Tumour size (cm)				0.027*	4.9123
>4	20	6	14		
≤4	20	13	7		
Histological grade				0.154	2.0301
I and II	32	17	15		
III and IV	8	2	6		
Lymph node metastasis				0.028*	4.8211
No	18	12	6		
Yes	22	7	15		

Note

Low/high by the sample median. Pearson's chi‐square test. *P* < 0.05 was considered statistically significant. * *P* < 0.05.

### 2.4 RNA isolation and quantitative real‐time PCR (qRT‐PCR)

Total RNA was extracted from ccRCC tissues and adjacent normal tissues or cells using RNA Isolator Total RNA Extraction Reagent (Vazyme, China) according to the manufacturer's protocol. Total RNA (1μg) was reverse‐transcribed to high‐quality cDNA using a PrimeScript RT Master Mix (Vazyme Biotech, Nanjing, China). qRT‐PCR was performed with the SYBR Green Mix (Vazyme Biotech). The expression of the miRNAs in this study was measured using the All‐in‐One™ miRNA qRT‐PCR Detection Kit (Vazyme Biotech, Nanjing, China). All qRT‐PCR assays were performed on an ABI 7500 System (Applied Biosystems, Foster City, CA, USA). The housekeeping gene glyceraldehyde 3‐phosphate dehydrogenase (GAPDH) or SNORD6 (U6 snRNA) was used as reference genes to normalize the expression levels of genes or miRNA. Data were analysed using the 2^‐ΔΔCt^ method to do the calculation by Applied Biosystems StepOnePlus Real‐Time PCR System (Applied Biosystems, USA). The primers for qRT‐PCR were listed as followed:

MIR4435‐2HG

Forward: 5’‐AATTTGCCACCACCCTGTGA‐3’,

Reverse: 5’‐ATGCCGTTTTAGGGGGACAG‐3’;

KLF6

Forward: 5’‐GGCCAAGTTTACCTCCGACC‐3’,

Reverse: 5’‐TAAGGCTTTTCTCCTTCCCTGG‐3’;

GAPDH

Forward: 5’‐GCTCTCTGCTCCTCCTGTTC‐3’,

Reverse: 5’‐ACGACCAAATCCGTTGACTC‐3’;

miR‐513a‐5p

Reverse transcriptional sequence:

5’‐GTCGTATCCAGTGCAGGGTCCGAGGTATTCGCACTGGATACGACATGACA‐3’,

Forward: 5’‐GCGCGTTCACAGGGAGG‐3’,

Reverse: 5’‐AGTGCAGGGTCCGAGGTATT‐3’;

U6

Forward: 5’‐CTCGCTTCGGCAGCACA‐3’,

Reverse: 5’‐AACGCTTCACGAATTTGCGT‐3’.

### 2.5 Cell transfection

To construct knockdown or overexpression of MIR4435‐2HG and miR‐513a‐5p in 769‐P or ACHN cells, respectively, sh‐MIR4435‐2HG, oe‐MIR4435‐2HG, miR‐513a‐5p mimic and miR‐513a‐5p inhibitor were used. The sequences of KLF6 were cloned into the plasmid pcDNA3.1 to form pcDNA3.1‐KLF6, and the small interfering RNA for KLF6 (si‐KLF6) was designed. The empty pcDNA3.1 and si‐NC were used as negative control. All products were obtained from GenePharma (Shanghai, China) and transfected into cells utilizing Lipofectamine 3000 (Invitrogen, Carlsbad, CA, USA) under manufacturer's protocols. The alterations in MIR4435‐2HG, miR‐513a‐5p and KLF6 were evaluated by qRT‐PCR before further analysis.

### 2.6 Cell proliferation assays

Cell proliferation was detected by the Cell Counting Kit‐8 (CCK‐8; Dojindo Laboratories, Kumamoto, Japan). 769‐P and ACHN cells were seeded in 96‐well plates at a density of 3000 cells/well and then cultured for 8, 24, 48 and 72 hours. Subsequently, 20 μL CCK‐8 was added to each well, the plates were incubated for 2 hours, and then the absorbance was measured at the wavelength of 450 nm.

### 2.7 Colony formation assays

Cells were transfected in a six‐well plate at a density of 200 cells/well. The cells were incubated for nearly 2 weeks at 37°C and 5% CO_2_ to fulfil the colony formation assay. Colonies were fixed with 4% paraformaldehyde, stained with 0.5% crystal violet and the number of colonies was counted using ImageJ.

### 2.8 Transwell assays

After matrigel (BD Biosciences, Shanghai, China) was added on the transwell chamber and clotted, cells (2 × 10^4^ cells per well) in 200 μL non‐serum medium were seeded into the top chambers. 500 μL medium containing 20% FBS was added to the bottom chamber. After 24 hours, the matrigel and the extra cells on the upper chamber were removed with cotton swab. The cells on the lower surface of the insert were then fixed with 4% paraformaldehyde for 20 minutes and stained for 20 minutes with 0.5% crystal violet. All images were taken under Quantity One software (Bio‐Rad, Hercules, CA, USA). All the results were calculated as the mean ± SD of three independent experiments.

### 2.9 TCGA analysis

The RNA‐seq data of ccRCC and matched normal samples were downloaded from The Cancer Genome Atlas (TCGA) Data Portal (https://tcga-data.nci.nih.gov/tcga/). Data quantification for the expression of miRNAs of MIR4435‐2HG was performed by the customized data analysis pipeline, including a series of steps that quality control, alignment and expression quantification. We used fold change ≥2.0 and a *P*‐value ≤ .05 as threshold to screen up‐regulated or down‐regulated genes.

### 2.10 Dual‐luciferase reporter assay

MIR4435‐2HG wild‐type (WT) containing miR‐513a‐5p–binding sites on the MIR4435‐2HG promoter region and MIR4435‐2HG mutant type (MUT) were ligated into PGLO vectors, respectively, named PGLO‐MIR4435‐2HG‐WT and PGLO‐MIR4435‐2HG‐MUT. Either PGLO‐MIR4435‐2HG‐WT or PGLO‐MIR4435‐2HG‐MUT was co‐transfected with miR‐513a‐5p mimc or NC plasmid into HEK‐293FT cells. After 24h, the cells were collected and lysed. In addition, the same conduction did on KLF6. Firefly and Renilla luciferase activities were measured with Dual‐Luciferase Reporter Assay System (Promega, Madison, WI, USA).

### 2.11 Co‐localization assays

To detect the location of MIR4435‐2HG and miR‐513a‐5p, we used Cy5‐labelled probes specific to MIR4435‐2HG and FAM‐labelled probes specific to miR‐513a‐5p (Servicebio, Wuhan, China). 769‐P cells were hybridized in hybridization buffer with the DNA oligo probes labelled with Cy5 for MIR4435‐2HG and FAM for miR‐513a‐5p. The images were acquired using a Leica SP5 confocal microscope (Leica Microsystems, Mannheim, Germany).

### 2.12 Western blotting

Total protein was prepared from 769‐P and Caki‐1 cells using RIPA buffer with a proteinase inhibitor and phosphatase inhibitors. The lysates were centrifuged on ice for 30 minutes and then centrifuged at 13400×g for 15 minutes at 4°C. The protein concentration was measured by a BCA Kit (Beyotime Biotechnology, Beijing, China). We separated the extracted proteins with SDS‐PAGE, which were transferred to PVDF membranes. 5% of non‐fat milk was used to block the membrane, which was incubated with primary antibodies against KLF6 at 4°C overnight. Subsequently, the membranes were incubated with the corresponding secondary antibody at room temperature for 1 hour. The expression of GAPDH was used as loading control. According to the manufacturer's instructions, a Chemiluminescence Reagent (ECL) Kit (Beyotime Biotechnology) was utilized to visualize protein bands. All antibodies were purchased from Servicebio (Wuhan, China).

### 2.13 Tumorigenicity assays in nude mice

The indicated stable cell lines (2 × 10^6^) were subcutaneously injected into the right flank of BALB/c (nu/nu) 4‐ to 6‐week‐old female nude mice. Tumour size was measured once per 4 days, and mice were killed to analyse the tumour burden after 4 weeks, and the tumour volume (V) was calculated using the formula: V = 1/2(length × width^2^). All procedures of animal experiments were performed in accordance with the Nanjing Medical University's Animal Ethics Committee.

### 2.14 Immunohistochemical

Haematoxylin and eosin (H&E) staining was utilized to select representative areas. Tissue samples embedded in paraffin were stained to identify and measure KLF6 levels. The tumours were detected with primary monoclonal probes for KLF6 overnight at 4°C. After incubation with a suitable second antibody, the tissue microarrays were treated with diaminobenzidine and counterstained with haematoxylin. Sections were visualized under a microscope (400× or 200×) (Olympus, Japan). The results were graded according to the percentage of positive cells.

### 2.15 Statistical analysis

The measurement data were expressed as the mean ± SD and were analysed in GraphPad Prism 7.0 (GraphPad Software, La Jolla, CA, USA). One‐way ANOVA analysis or two‐tailed Student's *t* tests were performed for *P*‐value analysis, as appropriate. The data of control group were chosen as y‐axis normalization controls in this manuscript. Unless otherwise noted, each experiment was carried out at least in triplicate. *P* < 0.05 was considered statistically significant.

## 3 RESULTS

### 3.1 Identification of MIR4435‐2HG as an lncRNA up‐regulated in ccRCC

**Figure 1:**
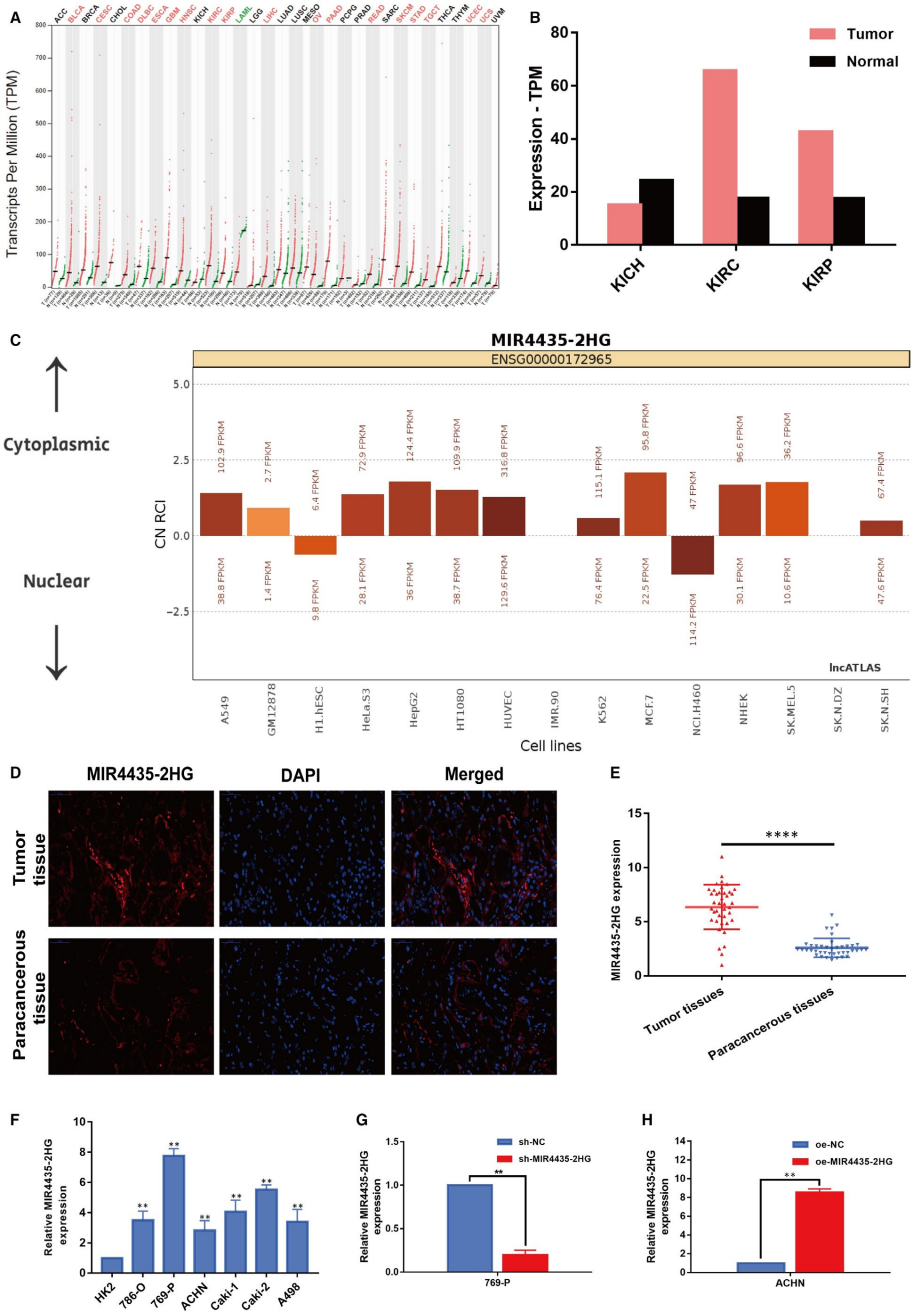
Identification of MIR4435‐2HG as an lncRNA up‐regulated in ccRCC. A‐B: GEPIA results of up‐expression of MIR4435‐2HG in ccRCC tissues. C: The subcellular localization of MIR4435‐2HG predicted on lncATLAS website. D: The subcellular localization of MIR4435‐2HG detected by FISH assay (400×). E: qRT‐PCR analysis of the expression levels of MIR4435‐2HG in 40 paired ccRCC tissues and the adjacent normal tissues. F: MIR4435‐2HG expression levels in ccRCC cell lines and human normal renal tubular epithelial cell line HK‐2 were detected by qRT‐PCR analysis. G‐H: The relative expression of MIR4435‐2HG determined by RT‐qPCR analysis following the treatment of knocking down MIR4435‐2HG in 769‐P cells (sh‐MIR4435‐2HG) or over‐expressing MIR4435‐2HG in ACHN cells (oe‐MIR4435‐2HG). All of the data were analysed from three independent experiments. * *P* < 0.05; ** *P* < 0.01;**** *P* < 0.001 vs control group

Initially, several online databases were used to probe the lncRNA‐mediated initiation and progression of ccRCC. Based on the analysis of gene expression profiling interactive analysis (GEPIA) data, we found that MIR4435‐2HG was highly expressed in various cancers (Figure 1A). What is more, significant up‐regulation of MIR4435‐2HG was identified in kidney renal clear cell carcinoma (KIRC) (Figure 1B), which was consistent with the results by analysing data from NCBI (https://www.ncbi.nlm.nih.gov/). As predicted by the lncATLAS website (http://lncatlas.crg.eu/), MIR4435‐2HG was mainly predicted to be localized in the cytoplasm, which was further confirmed by FISH (Figure 1C,D). To further investigate the role of MIR4435‐2HG in ccRCC tumorigenesis, a total of 40 ccRCC tissues and paracancerous normal tissues were collected from ccRCC patients to detect the expression level of MIR4435‐2HG. According to qRT‐PCR results shown in Figure 1E, ccRCC tissues exhibited higher levels of MIR4435‐2HG compared with paracancerous normal tissue. Moreover, MIR4435‐2HG was expressing in human normal renal tubular epithelial cell line HK‐2 and six ccRCC cell lines (786‐O, 769‐P, Caki‐1, Caki‐2, ACHN and A498) to varying degrees, among which 769‐P cells exhibited highest MIR4435‐2HG expression, whereas ACHN cells in the second (Figure 1F). To further analysis, we established knockdown model of MIR4435‐2HG with sh‐MIR4435‐2HG in human 769‐P cells and overexpression model of MIR4435‐2HG with oe‐MIR4435‐2HG in human ACHN cells (Figure 1G,H). Taken together, our findings revealed that MIR4435‐2HG levels were significantly high in ccRCC tissues and cell lines and it might be acted as an oncogene in ccRCC.

### 3.2 Long non‐coding RNA MIR4435‐2HG increased proliferation and invasion abilities of ccRCC cells

**Figure 2:**
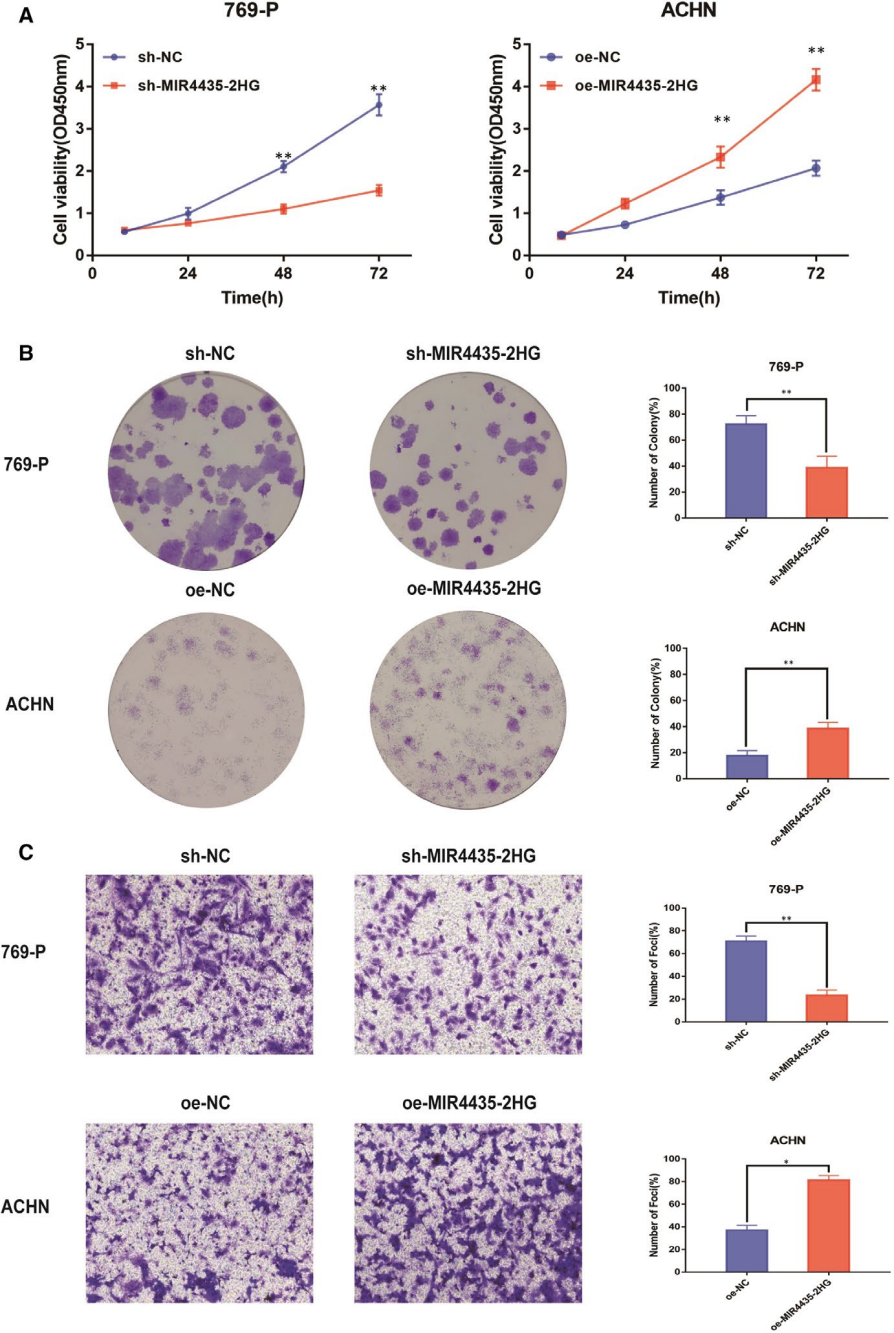
Long non‐coding RNA MIR4435‐2HG increased proliferation and invasion abilities of ccRCC cells. A: Cell proliferation was examined by CCK‐8 assays in sh‐MIR4435‐2HG group or oe‐MIR4435‐2HG group at the indicated time‐points. The shRNA control 769‐P cells or oe‐control ACHN cells were as control. B: Cell proliferation was determined by colony formation assay of impacts of MIR4435‐2HG in 769‐P and ACHN cells. C: Representative images revealing the invasion capacities of impacts of MIR4435‐2HG in 769‐P and ACHN cells. All of the data were analysed from three independent experiments. * *P* < 0.05; ** *P* < 0.01

To further investigate the biological effect of MIR4435‐2HG in ccRCC, we used 769‐P cells with sh‐MIR4435‐2HG and ACHN cells with oe‐MIR4435‐2HG. CCK‐8 assays elucidated that MIR4435‐2HG knockdown significantly inhibited cell growth ability in 769‐P cells, whereas MIR4435‐2HG overexpression notably increased it in ACHN cells (Figure 2A). Consistently, clone assay used for the cell proliferation detection showed the number of cell colonies in sh‐MIR4435‐2HG group was much smaller than that in sh‐NC groups, whereas in oe‐MIR4435‐2HG group, the number of cell colonies was significantly larger than that in oe‐NC groups. This fact highlighted that the overexpression of MIR4435‐2HG enhanced amplification of ccRCC cells (Figure 2B). Additionally, the regulation of MIR4435‐2HG had also clearly impacts on ccRCC cells invasion rate, which was measured by transwell assay. The results showed that the invasive ability was attenuated in 769‐P cells transfected with sh‐MIR4435‐2HG, whereas enhanced in ACHN cells transfected with oe‐MIR4435‐2HG (Figure 2C). Collectively, these results revealed that MIR4435‐2HG had tumour‐inductive activity in ccRCC progression.

### 3.3 MIR4435‐2HG interacted with miR‐513a‐5p and repressed its expression in ccRCC

In sequence, we attempted to obtain a better understanding of the mechanism of MIR4435‐2HG in promoting ccRCC progression. It has been known that competitive endogenous RNAs (ceRNAs), which sponge corresponding miRNAs to realize their modulating effects on target mRNAs, are the most well‐known mechanism of cytoplasmic lncRNAs,^18^ so we speculated that MIR4435‐2HG could regulate ccRCC through this way as well.

**Figure 3:**
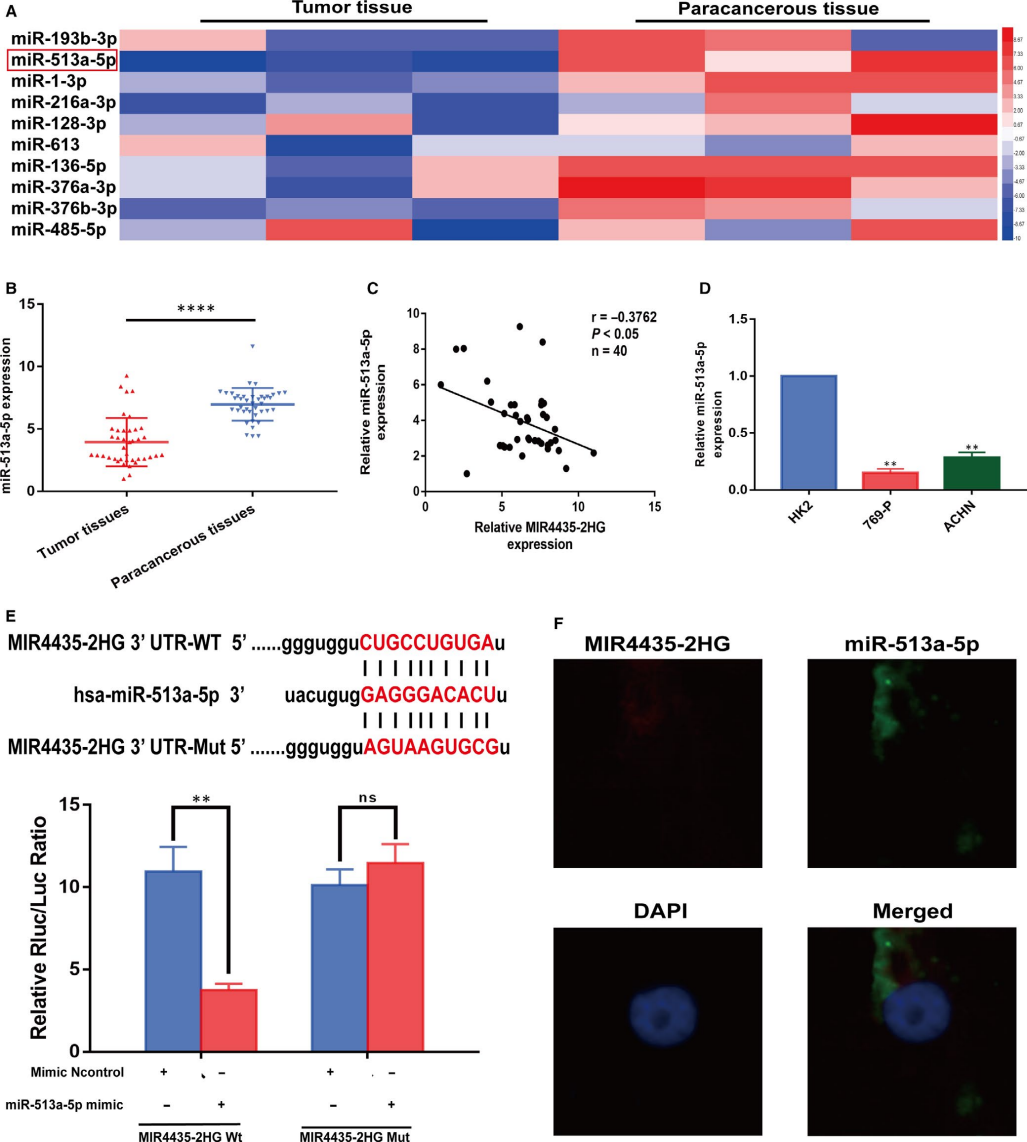
MIR4435‐2HG interacted with miR‐513a‐5p and repressed its expression in ccRCC. A: miRNA profiling analysis result of TCGA database showing abnormal downstream expressed target miRNAs of MIR4435‐2HG in paracancerous or ccRCC tumour tissues. miR‐513a‐5p is one of the most low‐expressed miRNAs in ccRCC tumour tissues. B: qRT‐PCR assay confirmed the relative expression of miR‐513a‐5p in 40 paired ccRCC cancer tissues compared with corresponding paracancerous normal tissues. C: Correlation between MIR4435‐2HG and miR‐513a‐5p expression in 40 ccRCC tumour tissues. D: miR‐513a‐5p expression level in ccRCC cell lines (769‐P and ACHN cells) and human normal renal tubular epithelial cell line HK‐2 was detected by qRT‐PCR assay. E: Schematic illustration of the predicted binding sites between MIR4435‐2HG and miR‐513a‐5p, and mutation of potential miR‐513a‐5p‐binding sequence in MIR4435‐2HG. Relative luciferase activities of wild‐type (WT) and mutated (MUT) MIR4435‐2HG reporter plasmid in human embryonic kidney (HEK) 293T cells co‐transfected with miR‐513a‐5p mimic. F: Co‐localization between MIR4435‐2HG and miR‐513a‐5p was observed by FISH. Nuclei were stained with DAPI. All of the data were analysed from three independent experiments. ** *P* < 0.01; *** *P* < 0.001; **** *P* < 0.001

Initially, we used TCGA database to further identify the target genes modulated by MIR4435‐2HG, among which miR‐513a‐5p was found significantly down‐regulated (fold change ≥2 or ≤0.5, *P* < 0.05) (Figure 3A). Furthermore, we confirmed the expression level of miR‐513a‐5p in ccRCC tissues by applying qRT‐PCR (Figure 3B). And we also validated the expression of MIR4435‐2HG and miR‐513a‐5p in 40 fresh ccRCC tissues and obtained a negative correlation between MIR4435‐2HG and miR‐513a‐5p (γ = −0.3762, *P *＜ 0.01) (Figure 3C). And the expression of miR‐513a‐5p in 769‐P and ACHN cells was down‐regulating compared with that in HK2 cells as we expected (Figure 3D). Furthermore, dual‐luciferase reporter gene assay was applied to assess whether MIR4435‐2HG acts on miR‐513a‐5p. The results showed that compared with NC group, the luciferase activity of cells transfected with miR‐513a‐5p mimic in MIR4435‐2HG‐WT group decreased significantly (*P* < 0.01), whereas there was no significant change in luciferase activity in cells transfected with miR‐513a‐5p mimic in MIR4435‐2HG‐MUT group (Figure 3E). We further performed co‐localization experiment to confirm the above results, which was consistent with the interaction of MIR4435‐2HG and miR‐513a‐5p (Figure 3F).

### 3.4 KLF6 was a direct target of miR‐513a‐5p in ccRCC progression

**Figure 4:**
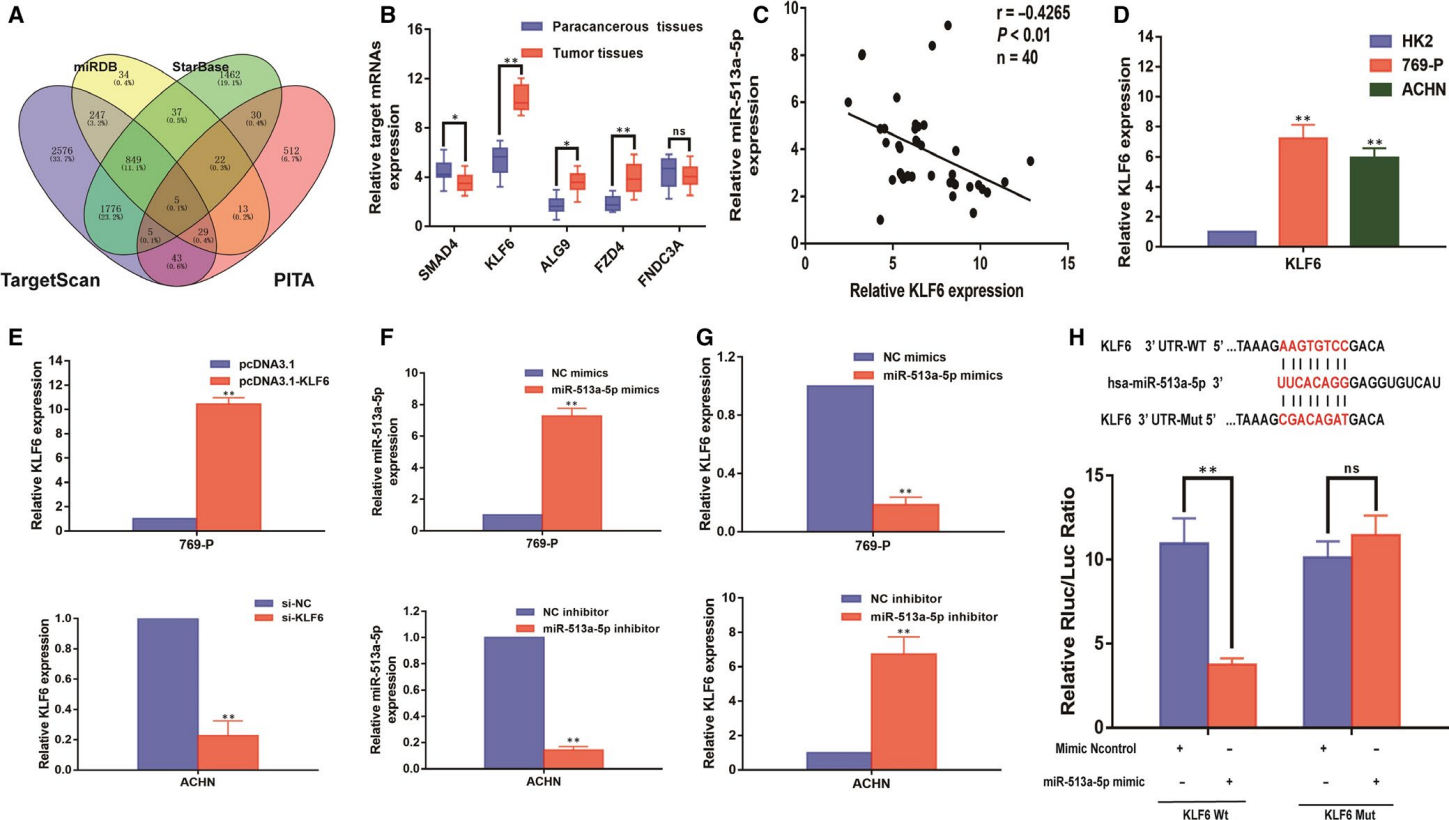
KLF6 was a direct target of miR‐513a‐5p in ccRCC progression. A: A schematic diagram used to search the target mRNAs of miR‐513a‐5p in four databases. B: qRT‐PCR assay confirmed the relative expression of five candidate target mRNAs of miR‐513a‐5p in 40 paired ccRCC cancer tissues and corresponding paracancerous normal tissues. C: Correlation analysis between KLF6 expression and miR‐513a‐5p expression in 40 ccRCC tumour tissues. D: Relative expression of KLF6 in ccRCC cell lines (769‐P and ACHN cells) and HK‐2 cells. E: Relative expression of KLF6 in 769‐P cells transfected with pcDNA3.1‐KLF6 and ACHN cells transfected with si‐KLF6. F: The relative expression of miR‐513a‐5p determined by qRT‐PCR analysis following the treatment of overexpressing miR‐513a‐5p in 769‐P cells (miR‐513a‐5p mimics) or knocking down miR‐513a‐5p in ACHN cells (miR‐513a‐5p inhibitor). G: Relative expression of KLF6 in 769‐P cells transfected with miR‐513a‐5p mimics and ACHN cells transfected with miR‐513a‐5p inhibitor. H: Schematic illustration of the predicted binding sites between miR‐513a‐5p and KLF6, and mutation of potential miR‐513a‐5p–binding sequence in KLF6. Relative luciferase activities of wild‐type (WT) and mutated (MUT) KLF6 reporter plasmid in human embryonic kidney (HEK) 293T cells co‐transfected with miR‐513a‐5p mimic. All of the data were analysed from three independent experiments. * *P* < 0.05; ** *P* < 0.01

To further explore the potential molecular mechanism of miR‐513a‐5p regulation in ccRCC, we collectively predicted potential conjugated mRNAs of miR‐513a‐5p with miRDB (http://www.mirdb.org/), StarBase (http://starbase.sysu.edu.cn/), TargetScan (http://www.targetscan.org/) and PITA (http://www.pita.org/) (Figure 4A). Then, we checked the expression of common mRNAs from these databases between ccRCC tissues and paracancerous normal tissues, indicating that KLF6 was dramatically increased in ccRCC tissues (Figure 4B). And as shown in qRT‐PCR assay, KLF6 was high expressing in 40 ccRCC patient tissues, which negatively related to miR‐513a‐5p expression level (γ = −0.4265, *P* < 0.01) (Figure 4C). Meanwhile, further verification of KLF6 expression was built in 769‐P and ACHN cells by qRT‐PCR (Figure 4D). And to verify the association between miR‐513a‐5p and KLF6, we first verified the transfection efficiency of overexpression of KLF6 with pcDNA3.1‐KLF6 in 769‐P cells and knockdown of KLF6 with si‐KLF6 in ACHN cells (Figure 4E). To further analysis, miR‐513a‐5p mimics were established in 769‐P cells and miR‐513a‐5p inhibitor was used in ACHN cells, and the results were verified by qRT‐PCR (Figure 4F). Then, we detected the expression of KLF6 in 769‐P and ACHN cells transfected with miR‐513a‐5p mimics or miR‐513a‐5p inhibitor, and the results revealed that miR‐513a‐5p could regulate the expression of KLF6 (Figure 4G). Finally, we performed luciferase reporter assays by co‐transfecting luciferase reporter plasmids with miR‐513a‐5p. The overexpression of miR‐513a‐5p decreased the luciferase activity driven by KLF6‐WT in HEK293FT cells, but did not change the activity of KLF6‐MUT, which suggested that KLF6 was a direct target of miR‐513a‐5p (Figure 4H).

### 3.5 miR‐513a‐5p inhibited proliferation and invasion abilities of ccRCC cells

**Figure 5:**
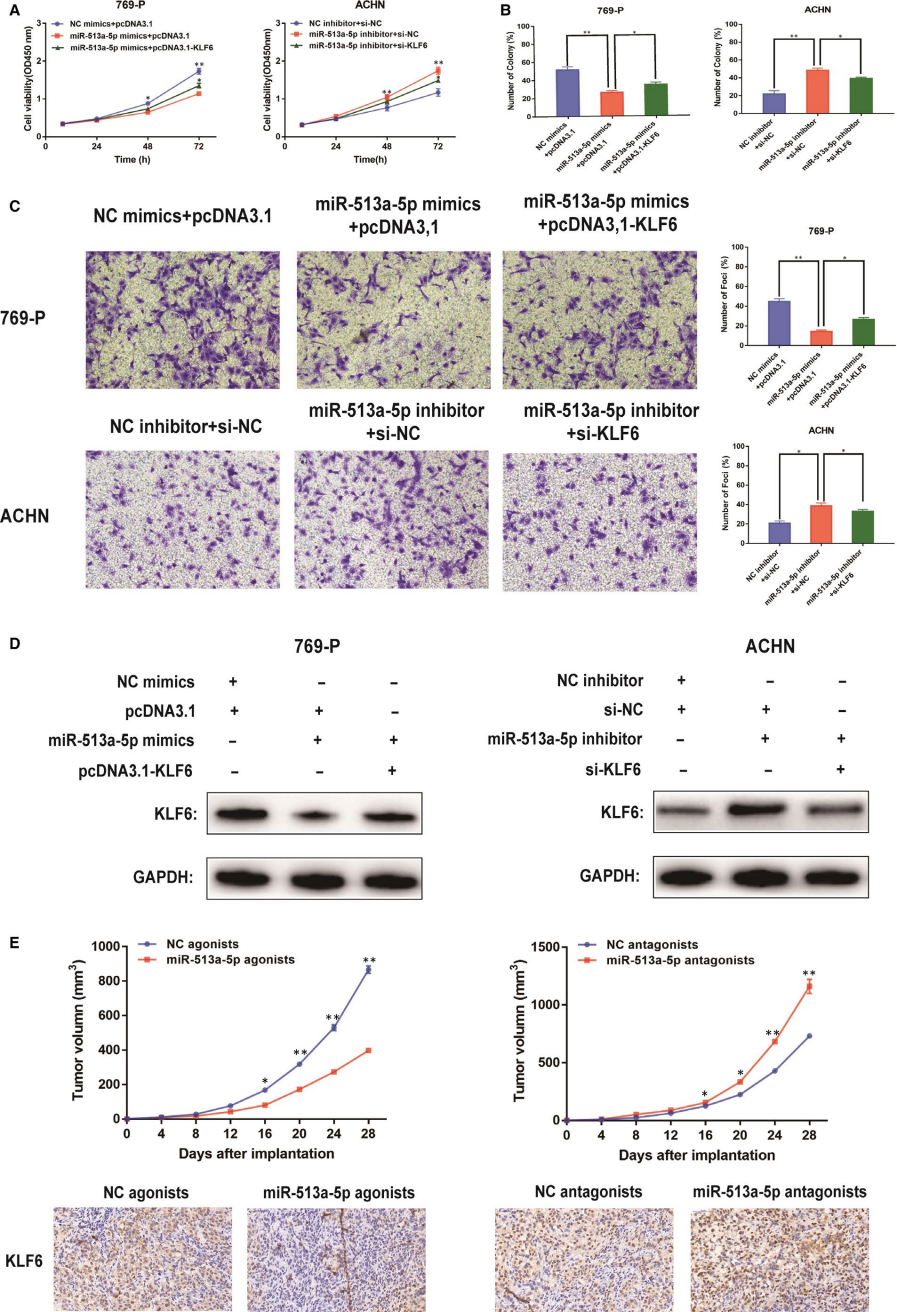
miR‐513a‐5p inhibited proliferation and invasion abilities of ccRCC cells. A: Cell proliferation was examined by CCK‐8 assays in miR‐513a‐5p mimics+pcDNA3.1‐KLF6 group or miR‐513a‐5p inhibitor+si‐KLF6 group at the indicated time‐points. The NC cells were as control. B: Cell proliferation was determined by colony formation assay of impacts of miR‐513a‐5p and KLF6 in 769‐P and ACHN cells. C: Representative images revealing the invasion capacities of impacts of miR‐513a‐5p and KLF6 in 769‐P and ACHN cells. D: Western blotting results of KLF6 in different transfected groups of 769‐P and ACHN cells. E: Tumour volume of the xenograft in each group. The tumour sections from different transfected groups of xenograft mouse models were subjected to immunohistochemistry staining using antibodies against KLF6 (400×). All of the data were analysed from three independent experiments. * *P* < 0.05; ** *P* < 0.01

To elaborate the function of miR‐513a‐5p in proliferation and invasion abilities of ccRCC cells, the experiments in vitro were conducted. Different transfection groups were constructed in 769‐P and ACHN cells. The up‐regulation of KLF6 expression in ccRCC cells could reverse the promotion on cell viability (Figure 5A,B) and invasive capacity (Figure 5C) caused by miR‐513a‐5p up‐expression. Accordingly, the down‐regulation of KLF6 could reverse inhibition on cell viability (Figure 5A,B) and invasive capacity caused by miR‐513a‐5p knockdown Figure 5C). In addition, KLF6, the target gene of miR‐513a‐5p, in different transfection groups of 769‐P and ACHN cells, was detected by Western blot, and the results are displayed in Figure 5D. Moreover, tumours of miR‐513a‐5p antagonists groups presented higher positive rate of KLF6 compared with NC antagonist groups (Figure 5E). These results highlighted that miR‐513a‐5p could suppress tumorigenesis of ccRCC by down‐regulating KLF6.

### 3.6 MIR4435‐2HG promoted tumours in ccRCC cells via modulating KLF6

**Figure 6:**
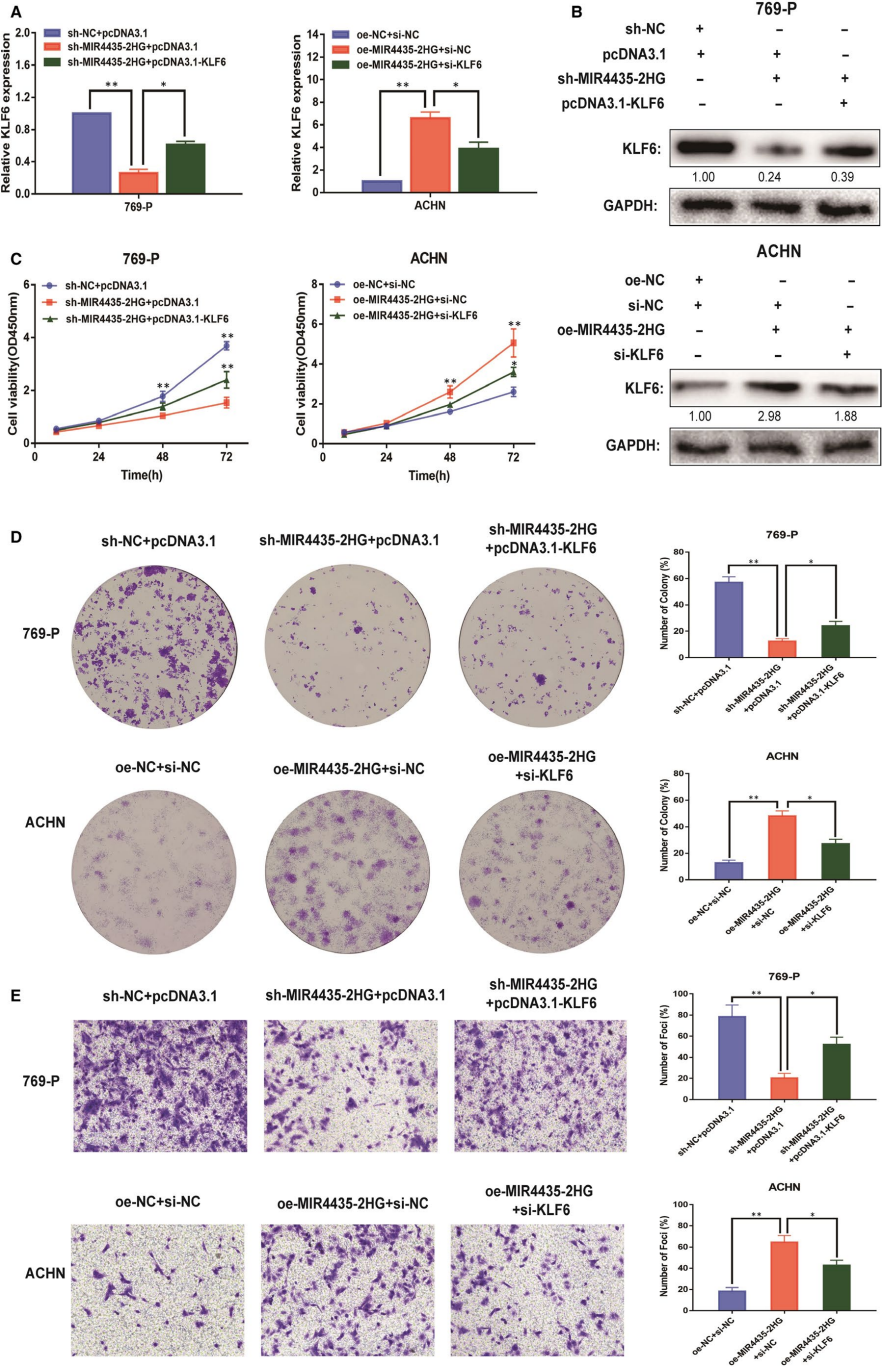
MIR4435‐2HG enhanced the proliferation and invasion abilities of ccRCC cells by regulating KLF6. A: qRT‐PCR was conducted to verify the relative expression of KLF6 in 769‐P transfected with NC, sh‐MIR4435‐2HG, sh‐MIR4435‐2HG+pcDNA3.1‐KLF6 and ACHN cells transfected with NC, oe‐MIR4435‐2HG and oe‐MIR4435‐2HG+si‐KLF6. B: The expression of KLF6 was analysed by Western blotting with the indicated antibodies and samples from the 769‐P and ACHN cells in different transfected groups. C: CCK‐8 assay of 769‐P and ACHN cells in different transfected groups. D: Cell proliferation was determined by colony formation assay of different transfected groups in 769‐P and ACHN cells. E: Representative images revealing the invasion capacities of impacts of different transfected groups in 769‐P and ACHN cells. All of the data were analysed from three independent experiments. * *P* < 0.05; ** *P* < 0.01

The KLF6 expression of 769‐P and ACHN cells in different transfection groups was detected by qRT‐PCR, and the results are displayed in Figure 6A. Compared with sh‐NC+pcDNA3.1 group, the expression level of KLF6 was notably reduced in sh‐MIR4435‐2HG+pcDNA3.1 group of 769‐P cells, whereas markedly increased in sh‐MIR4435‐2HG+pcDNA3.1‐KLF6 group of 769‐P cells. Meanwhile, compared with oe‐NC+si‐NC group, the expression level of KLF6 was significantly increased in oe‐MIR4435‐2HG+si‐NC group of ACHN cells, whereas clearly decreased in oe‐MIR4435‐2HG+si‐KLF6 group of ACHN cells. The results were also verified in Western blot (Figure 6B). And functional experiment, such as CCK‐8 assay, clone assay and transwell assay, also performed in 769‐P and ACHN cells of different transfection groups (Figure 6C‐E). According to the result, we could reveal that the impact of KLF6 expression was able to reverse the opposite impact of MIR4435‐2HG expression. These results suggested up‐regulation of MIR4435‐2HG could lead to tumorigenesis promotion of ccRCC through up‐regulating KLF6.

### 3.7 Decreasing MIR4435‐2HG contributed to suppress tumorigenesis and tumour progression by down‐regulating KLF6 in vivo

**Figure 7:**
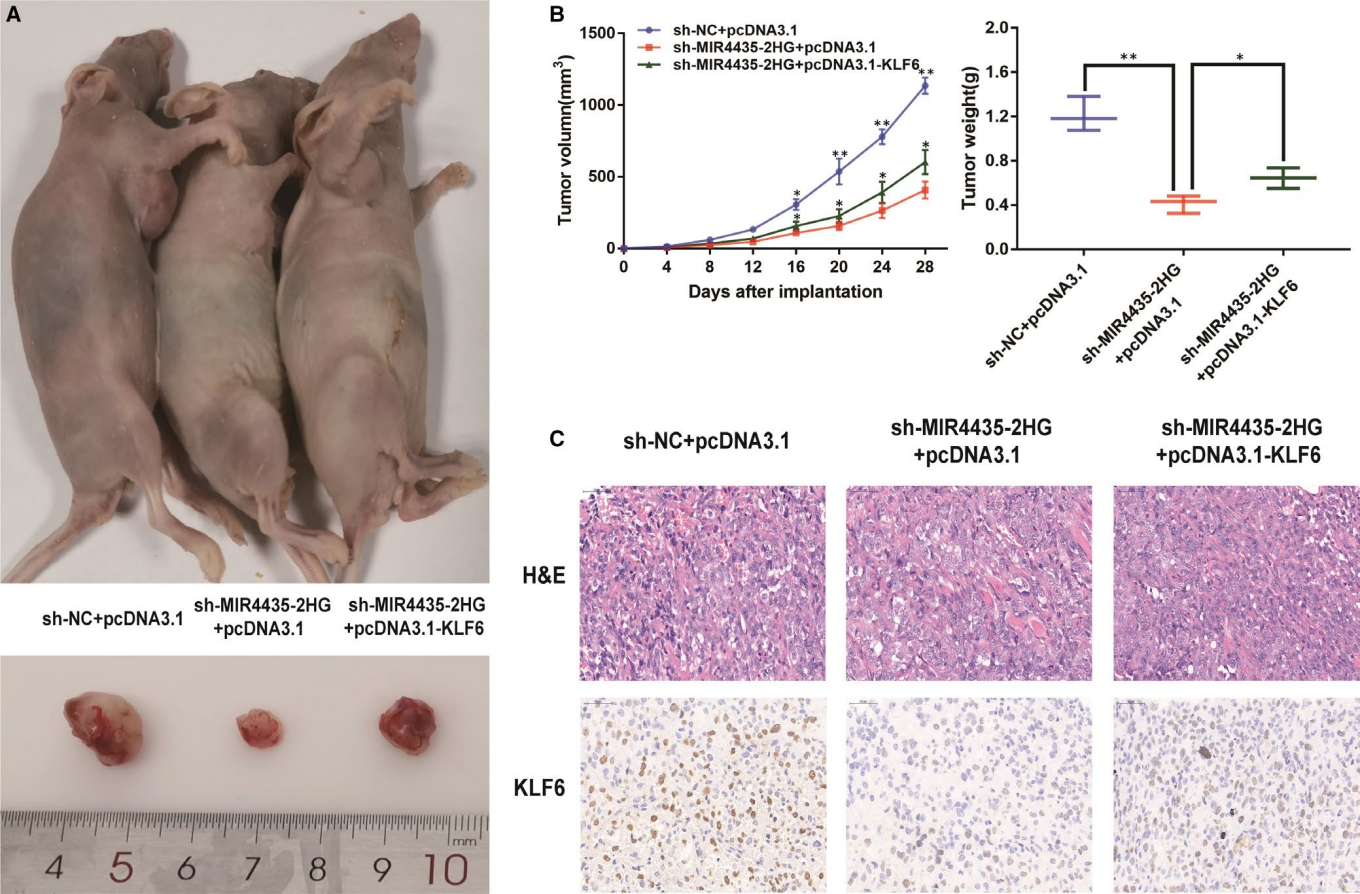
Decreasing MIR4435‐2HG contributed to suppress tumorigenesis and tumour progression by down‐regulating KLF6 in vivo. A: Representative images of the xenograft tumours in subcutaneous xenograft mouse model injected with 769‐P cells transfected with NC, sh‐MIR4435‐2HG and sh‐MIR4435‐2HG+pcDNA3.1‐KLF6. B: Tumour volume and weight of the xenograft in each group. C: The tumour sections from different transfected groups of xenograft mouse models were subjected to H&E staining and immunohistochemistry staining using antibodies against KLF6 (400×). All of the data were analysed from three independent experiments. * *P* < 0.05; ** *P* < 0.01

Figure 7 was conducted for the purpose of exploring the roles of MIR4435‐2HG and KLF6 in tumour growth in vivo. MIR4435‐2HG knockdown caused less tumour formation and significantly decreased tumour size compared with sh‐NC+pcDNA3.1 group, whereas results demonstrated that the effect of MIR4435‐2HG knockdown on tumour growth was reversed by KLF6 overexpression (Figure 7A,B). Consistent with the previous results, immunochemistry analysis confirmed that KLF6 proliferation index in sh‐MIR4435‐2HG+pcDNA3.1‐xenografted tumours was weaker than that in sh‐NC+pcDNA3.1‐xenografted tumours. Moreover, tumours of sh‐MIR4435‐2HG+pcDNA3.1‐KLF6 groups presented higher positive rate of KLF6 compared with sh‐MIR4435‐2HG+pcDNA3.1 groups (Figure 7C). These results highlighted the inhibition of MIR4435‐2HG could suppress tumorigenesis and tumour growth of ccRCC in vivo by decreasing KLF6.

## 4 DISCUSSION

RCC is well‐recognized malignancies of the urinary system, which accounts for 4% of adult malignancies, and annual estimates of newly diagnosed cases are in rising trend year by year steadily.^24^ About 70% of ccRCC patients have been diagnosed clinically localized disease, and the mainstay of treatment is surgical resection; however, post‐operative metastatic diseases occur in 20%‐30% of ccRCC patients.^25^ So, because of the unsatisfaction of relatively poor detection and prognosis, numerous researches on tumour treatment‐related factors have been receiving increasing attention.^24^, ^25^ It has been proved that lncRNAs have been clarified by growing evidence as active biological molecules, which played an extensive modulatory role in ccRCC carcinogenesis and progression.^26^, ^27^, ^28^, ^29^ According to the location, lncRNAs could realize their function through implicating in the genesis and development of target genes at different levels. For example, lncRNA DILC suppressed the proliferation and invasion of renal cancer cells at epigenetic level through repressing the PTEN ubiquitination in the cytoplasm.^30^ And in the nucleus, lncRNAs HOXA11‐AS could exert their function through regulating MMP16 expression.^31^ In tumour biology, MIR4435‐2HG was typically up‐regulated in malignant tissues in gastric cancer, glioblastoma and hepatocellular carcinoma.^21^, ^22^, ^23^ In the present study, we identified the lncRNA MIR4435‐2HG as a highly overexpression lncRNA in ccRCC. Nevertheless, the molecular mechanisms and potential function of MIR4435‐2HG in ccRCC were still unclear.

Initially, the key findings in our study were that the expression of MIR4435‐2HG was significantly up‐regulated in ccRCC tissues relative to corresponding paracancerous normal tissues from TCGA analysis, which was in compliance with the result of clinical ccRCC specimens. Moreover, we characterized the tumorigenic role of MIR4435‐2HG in ccRCC cells by gain‐ and loss‐of‐function assays. In vitro, the results shed light that knocking down MIR4435‐2HG in 769‐P cells would significantly inhibit cell proliferation and invasion. In contrast, overexpression of MIR4435‐2HG in ACHN cells robustly increased ccRCC cell proliferation and invasion. Therefore, our investigations shed a lot of light on MIR4435‐2HG severed as an oncogene in ccRCC and could be explored as a potential diagnostic and treatment indicator for ccRCC.

In the subsequent study, we aimed to uncover the potential mechanism of how MIR4435‐2HG affected tumorigenesis and development of ccRCC. lncRNAs exert their functions may depend on where they are.^32^ In the cytoplasm, lncRNAs may function as decoys for miRNAs and their target genes,^33^ whereas in the nucleus, lncRNAs may be involved in guiding RNA‐binding protein of transcription factors or epigenetic regulation.^34^ In our study, we revealed that MIR4435‐2HG was mainly localized in the cytoplasm but not in the nucleus, indicating its potential to regulate the expression of downstream gene through the way of ‘decoy’.

In our investigation, bioinformatic analysis showed that miR‐513a‐5p bound to MIR4435‐2HG, which was confirmed by qRT‐PCR and dual‐luciferase reporter assay. To further explore the potential conjugated mRNAs of miR‐513a‐5p, we collected the data from MiRDB, StarBase, TargetScan and PITA and found that KLF6 was included in the intersection of the databases. KLF6, also called Kruppel‐like factor 6, was demonstrated to enhance the activity of mRNAs that maintain carcinogenic transcriptional networks in ccRCC. For instance, Syafruddin SE et al revealed that KLF6 supported the expression of lipid metabolism genes and promoted the expression of PDGFB, which enhanced transcriptional networks by activating mTOR signalling.^35^ Bioinformatic analysis showed that miR‐513a‐5p bound to KLF6, which was also confirmed by qRT‐PCR and dual‐luciferase reporter assay. The results revealed that miR‐513a‐5p could interact with KLF6, therefore modulated the expression of KLF6.

To demonstrate that the axis that MIR4435‐2HG promoted KLF6 expression by sponging to miR‐513a‐5p in ccRCC, we designed a rescue experiment to reveal the role of MIR4435‐2HG/miR‐513a‐5p/KLF6 axis in ccRCC. The results showed that the change in KLF6 expression could reverse the function of MIR4435‐2HG on ccRCC in vitro and in vivo. These findings provided the evidence that MIR4435‐2HG was a potential diagnostic biomarker and a critical molecular target for tumour progression for ccRCC.

## 5 CONCLUSION

In conclusion, our study illustrated that lncRNA MIR4435‐2HG could serve as an oncogene in ccRCC by facilitating ccRCC cell proliferation and invasion. Mechanistically, we found that MIR4435‐2HG directly sponged miR‐513a‐5p, which was down‐regulated in ccRCC, and promoted KLF6 expression by disrupting the interaction between miR‐513a‐5p and KLF6 in ccRCC. Together, characterization of the MIR4435‐2HG/miR‐513a‐5p/KLF6 axis might provide innovative insights into the prevention and treatment progress for ccRCC.

## CONFLICT OF INTEREST

The authors declare no conflicts of interest that pertain to this work.

## AUTHOR CONTRIBUTIONS

All authors have read and approved the final manuscript.

## Data Availability

The data that support the findings of this study are available from the corresponding author upon reasonable request.
